# Thoracic sarcopenia as a comorbidity-independent predictor of length of stay in congenital cardiac surgery

**DOI:** 10.21203/rs.3.rs-6234626/v1

**Published:** 2025-03-27

**Authors:** Christopher E. Greenleaf, Julija Dobrila, Blaz Podgorsek, Martin Osorio Nader, Anh V. D. Nguyen, Santosh Uppu, Wen Li

**Affiliations:** Houston Children’s Heart Institute, Memorial Hermann Hospital, University of Texas Health McGovern Medical School, Houston, TX, USA; Houston Children’s Heart Institute, Memorial Hermann Hospital, University of Texas Health McGovern Medical School, Houston, TX, USA; Houston Children’s Heart Institute, Memorial Hermann Hospital, University of Texas Health McGovern Medical School, Houston, TX, USA; Houston Children’s Heart Institute, Memorial Hermann Hospital, University of Texas Health McGovern Medical School, Houston, TX, USA; Houston Children’s Heart Institute, Memorial Hermann Hospital, University of Texas Health McGovern Medical School, Houston, TX, USA; Houston Children’s Heart Institute, Memorial Hermann Hospital, University of Texas Health McGovern Medical School, Houston, TX, USA; Clinical and Translational Sciences, University of Texas Health McGovern Medical School, Houston, TX, USA

**Keywords:** thoracic sarcopenia, congenital cardiac surgery, skeletal muscle mass, postoperative outcomes, frailty in pediatric surgery, computed tomography muscle assessment

## Abstract

**Objective:**

Thoracic sarcopenia, as shown by reduced thoracic skeletal muscle volume (TSMV) on imaging, predicts adverse outcomes after surgery in other patient populations. We sought to ascertain whether a decrease in the thoracic muscle volume serves as a prognostic indicator for postoperative morbidity and mortality in patients undergoing surgery for congenital cardiac anomalies.

**Methods:**

All consecutive patients who underwent an index congenital cardiac operation were retrospectively analyzed. Chest cross-sectional imaging within 6 months preoperatively was identified. The TSMV was calculated at the T6 to T10 thoracic vertebrae level. Patients were stratified into high and low muscle groups using the median of muscle cross-sectional volume.

**Results:**

101 patients were included. Those with low TSMV were more likely to be less than one year old, had lower body weight, and had more preoperative comorbidities than those with high thoracic muscle volume. In univariate analysis, patients with low TSMV had a longer hospital length of stay (LOS) (10 vs. 7 days, p = 0.01) and more risk of hospital mortality (10.2% vs. 0%, p = 0.024). In the multivariable models, low thoracic volume showed no clear association with overall complications, cardiopulmonary complications, or intubation duration. Higher TSMV did predict a shorter LOS (MD per 10,000 mm^3^ increase: −70.7 days, CI −12.7 – −1.4, P = 0.01).

**Conclusions:**

Our findings indicate that thoracic sarcopenia holds an association with LOS and mortality in patients undergoing surgery for congenital cardiac anomalies. As such, thoracic sarcopenia merits consideration as a potential risk factor in the preoperative assessment of patients presenting for congenital cardiac surgical interventions.

## Introduction

Over the last 50 years, the operative mortality for congenital heart surgery has significantly decreased [1]. However, despite advancements in perioperative care, patients still experience considerable morbidity [2]. Effective prediction of postoperative complications remains limited, requiring detailed evaluations of operation complexity, patient comorbidities, age, size, physiologic status, and urgency of the procedure [3].

Historically, low muscle mass, an indicator of frailty, has been linked to poor outcomes after surgery [4]. Computed tomography (CT) has become a reliable tool for measuring skeletal muscle mass and function by assessing muscle area [5]. While diminished muscle area has been correlated with increased postoperative morbidity and mortality in surgical fields, these studies predominantly centered on adult populations and relied on specific abdominal CT slices for assessment [6–8].

Routine use of thoracic cross-sectional imaging in patients undergoing congenital heart surgery allows for readily available assessment of thoracic musculature. The utility of thoracic muscle assessment and correlation with postoperative outcomes has varied across different surgical specialties [9, 10].

We hypothesize that thoracic sarcopenia may serve as an indicator of reduced cardiopulmonary function, poor nutritional status, congestive heart failure, or overall frailty, leading to increased perioperative complications and mortality. To test this hypothesis, this study will correlate the postoperative outcomes after congenital cardiac surgery in patients depending on their thoracic skeletal muscle volume (TSMV).

## Materials and Methods

The prospectively collected Children’s Memorial Hermann Hospital (CMHH) Society of Thoracic Surgeons (STS) database was retrospectively analyzed for patients with congenital heart disease (CHD) who underwent an STS index operation between January 2009 and February 2020. Inclusion criteria required CHD, an index congenital cardiothoracic operation, and a thoracic CT or magnetic resonance imaging (MRI) performed within six months prior to the operation. Patients were excluded if chest cross-sectional imaging was not obtained within 6 months preoperatively, the chest wall muscles were incompletely imaged, or clinical data was unavailable. UTHealth McGovern Medical School investigational review boards approved the study, with informed consent waived (IRB #HSC-MS-20–1145).

The primary outcome was a composite of cardiorespiratory complications that included mortality, cardiac and pulmonary complications, unplanned reoperations, 30-day readmissions, and intubation lasting > 48 hours. Secondary outcomes included mortality, perioperative complications, reoperations, readmissions, hospital length of stay (LOS), and individual system complications. Postoperative complications were defined per STS Congenital Heart Surgery Database criteria. Preoperative, operative, and follow-up data were collected from electronic medical records.

## Thoracic skeletal muscle volume

**Figure 1:**
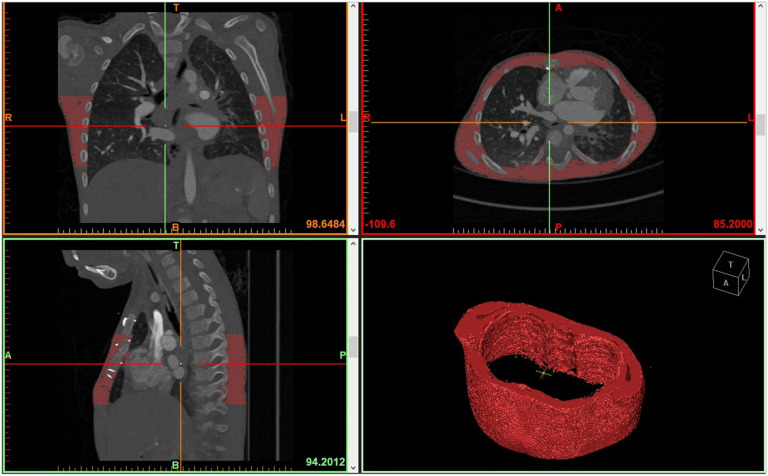
Picture of thoracic musculature cross sectional imaging used to segment the thoracic skeletal musculature to estimate skeletal muscular area

Preoperative chest CT or MRI obtained for routine clinical care within 6 months were analyzed on a research workstation. TSMV at the T6–T10 levels was calculated by an analyst (J.D.) using threshold segmentation [−29 to 150 Hounsfield units (HU)] and Mimics inPrint version 3.0 (Materialise NV, Leuven, Belgium). Figure 1 shows a 3D segmentation of thoracic musculature. Initially, the entire thoracic skeletal musculature from T1 to T10 was analyzed, but the variable inclusion of the shoulder musculature in the imaging introduced unacceptable variability. Therefore, the analysis was refined to focus on the T6 to T10 levels. A board-certified pediatric cardiologist with expertise in imaging (S.U.) verified segmentation accuracy in 10% of randomly selected cases. Analysts were blinded to clinical outcomes. Patients ≥ 12 years old were stratified by sex, while those < 12 years were not, assuming minimal pre-pubertal sex differences. Muscle volumes was analyzed as both a continuous and categorical variable, with low muscle area defined as values below the cohort median.

## Statistical Analysis

Categorical variables were summarized as frequencies and proportions, while continuous variables were reported as medians with interquartile ranges. Wilcoxon test and Fisher’s exact test were used for two-group comparisons of continuous and categorical variables, respectively. Multivariable linear and logistic regression, adjusted for potential confounders (age, weight, and preoperative factors), evaluated the effect of TSMV on LOS, complications, cardiopulmonary issues, intubation duration, 30-day readmissions, unplanned re-interventions, and mortality. STAT scores were dichotomized (1–2 and 3–5) for multivariable regression analysis. Multivariable models were limited by the insufficient number of events in some outcome variables. For LOS and intubation duration, the sandwich formula was applied to address skewed distributions and calculate robust variances and P-values.

In multivariable regression models, TSMV was treated as a continuous variable, with interactions between volume and age included. If the interaction effect was significant, subgroup analyses for each age group were added to assess volume as an independent risk factor and its effect by age. If the interaction effect was insignificant, the term was excluded. Inter- and intra-reader agreement was evaluated using the intra-class correlation coefficient. Due to high correlations (rho = 0.89) between height, weight, and age, only weight was included in the analysis. Statistical significant difference was defined as P-value < 0.05, and analyses were performed using R version 4.0.5.

## Results

**Figure 2:**
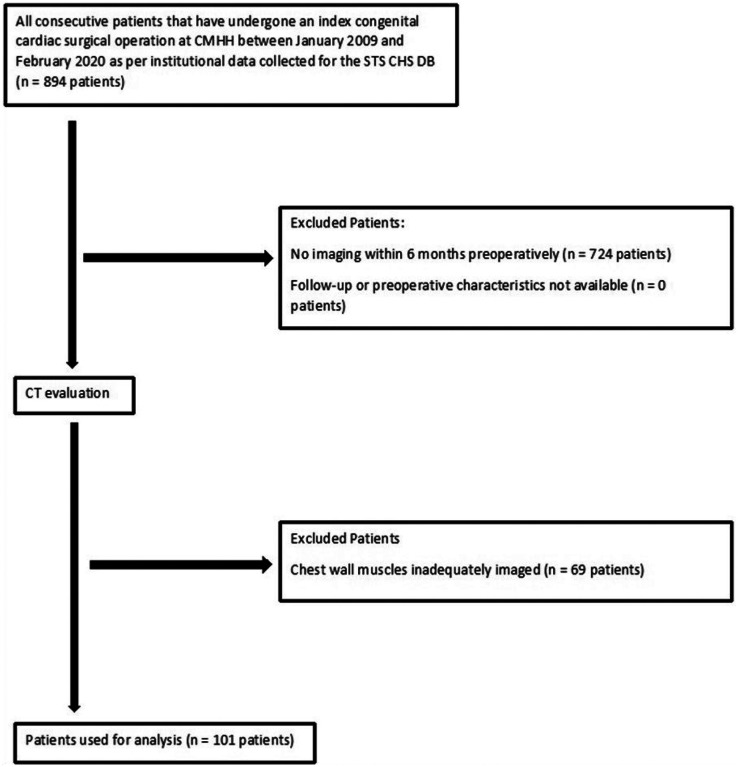
Flow chart of patient inclusion. CHS = congenital heart surgery, CMHH = Children’s Memorial Hermann Hospital, CT = computed tomography, DB = database, STS = Society of Thoracic Surgeons

Between 2007 and 2021, there were 894 patients with congenital heart disease who underwent an STS index cardiothoracic operation at our center. Figure 2 shows the inclusion and exclusion pathway for the patients. 724 patients (81.0%) were excluded because there was no CT or MRI of the chest within 6 months prior to the operation. After imaging evaluation, of the 170 patients, 69 patients (40.5%) were excluded because the chest wall skeletal muscles were inadequately imaged to give a reliable analysis. This left 101 patients (11.3%) of the original 894 patients for final analysis to be included in the study. There were 52 patients (51%) in the high TSMV group and 49 patients (49%) in the low TSMV group. The three most common index operations were bidirectional Glenn anastomosis (n = 10, 9.9%), partial anomalous pulmonary venous return repair (n = 9, 8.9%), and Fontan (n = 6, 5.9%). The operation was performed by sternotomy in 94 patients (93.1%) and thoracotomy in 7 patients (6.9%).

Excellent intra- and inter-class agreement was achieved with intraclass correlation coefficients of 0.998 (95% CI 0.994 to 1) and 0.993 (95% CI 0.975 to 0.998), respectively.

Most patients were between 1 and 18 years of age (58.4%). Patients < 12 years old had a median sum of the TSMV of 169,874 m^3^. Male patients ≥ 12 years old had a median sum of the TSMV of 1,672,050 m^3^, while female patients ≥ 12 years old had a median sum of the TSMV of 1,023,580 m^3^. Table 1 shows the descriptive statistics of the included patients compared between low and high thoracic muscle groups. Compared with the low muscle group, patients in the high muscle group were significantly more likely to be older than 1 year of age [number (%) of infant: 2 (3.8%) vs. 29 (59.2%); P = 0.001], weighed more [17.1 kg (12.4, 27.0 kg) vs. 8.0 kg (6.6, 10.1); P = 0.001], were taller (103.5 cm (91.0, 128.2) vs. 70.0 (65.0, 79.5); P = 0.001], and had less of an incidence of any preoperative comorbidity [0.0 (0.0, 1.0) vs. 1.0 (0.0, 2.0), P = 0.018]. There was no significant difference with regard to gender, prematurity, individual comorbidities, genetic syndromes, STAT operative risk stratification, or cardiopulmonary bypass length.

**Figure 3:**
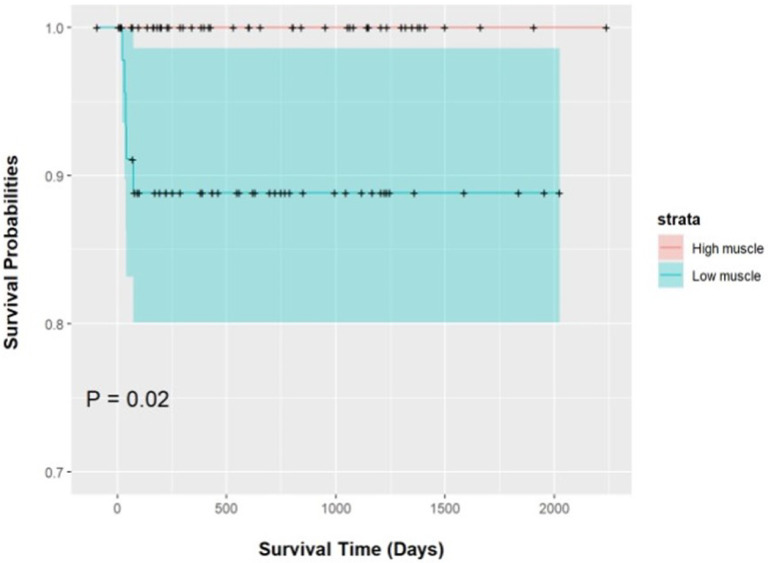
Kaplan Meier curves for survival between patients with low vs high thoracic musculature

The univariable analysis of outcomes comparing the high and low muscle groups is described in Table 2. Patients in the low group tended to have longer LOS [10 days (7, 22 days) vs. 7 days (4, 16 days); P = 0.011] and were significantly more likely to have an in-hospital mortality [5 (10.2%) vs 0 (0%); P = 0.024]. Figure 3 is the Kaplan-Meier survival curve. There was no difference in the composite outcomes of STS-categorized episode of care complications and mortality (P = 0.315) or the composite STS-derived cardiorespiratory complications and mortality (P = 0.691). There was also no difference in the incidence of individual organ system complications.

Table 3 shows the multivariable analysis of association of continuous TSMVwith LOS, any complication, cardiopulmonary complications, and intubation duration when controlled for variables with statistical significance on univariate analysis. These control variables included age, weight, and preoperative comorbidity. 30-day readmissions, unplanned reinterventions, and mortality were not applicable for a regression model because there were so few events (n = 8, 8, and 5, respectively). In the multivariable models, no clear association was seen with TSMV and any complication (odds ratio [OR] per 10000 mm^3^ increase: 1.01, CI 0.98–1.04, P = 0.58), cardiopulmonary complications (OR per 10,000 mm^3^ increase: 1.00, CI 0.97–1.03, P = 0.94) or intubation duration (mean difference [MD] per 10,000 mm^3^ increase: 0.08, CI −0.25–0.41, P = 0.65). However, higher TSMV was associated with a significantly shorter LOS (MD per 10,000 mm^3^ increase: −70.7 days, CI −12.7 – −1.4, P = 0.01).

## Discussion

Thoracic sarcopenia, defined as low TSMV segmented from preoperative routine thoracic cross-sectional imaging, is independently associated with LOS after congenital cardiac surgery. This holds true in an analysis accounting for variables with known association with poor outcomes, including age, weight, and preoperative non-cardiac comorbidities. We also found that thoracic sarcopenia was associated with operative mortality on univariable analysis (10.2% vs. 0%, P = 0.024).

Regrettably, there is a paucity of research on the preoperative assessment of skeletal muscle as a predictor of postoperative risk in pediatric populations. In a study involving pediatric patients with liver cirrhosis awaiting transplant, Jitwongwai S, et al. found that indexed psoas muscle area correlated with waitlist mortality and post-transplant outcomes. However, this association was not evident in multiple logistic regression analyses. Notably, patients with diminished muscle area experienced a prolonged LOS post-transplantation compared to those with a more substantial muscle area [11]. This observation aligns with other studies, including our own, which have shown skeletal muscle to be a reliable predictor of LOS, though its efficacy in predicting other outcomes remains inconsistent [12]. This inconsistency may arise from the lack of standardized metrics for skeletal muscle assessment in children, including the challenges of comparing rapidly changing body compositions across age groups, varied definitions of low muscle area or volume, and the complex physiology of the perioperative period. A scoping review by Metzger et al. on sarcopenia and outcomes in pediatric surgical patients revealed a staggering diversity in evaluation methods and definitions, including 5 different evaluation modalities and 15 different definitions of sarcopenia among 20 different manuscripts [13].

Children often lack the capacity to engage in assessments of frailty designed for older individuals, such as a 6-minute walk test or hand grip strength. Improving or utilizing cross-sectional imaging in novel ways may help to predict frailty in a wide array of diagnoses. There are several ways that could be used to improve this including standardizing the measurements of body composition valuation within pediatric patients similar to cardiac structure z-scores or using better imagistic assessments of functional frailty within a patient’s body. For instance, muscle density, which reflects muscle quality rather than mere size, might offer more insights. Furzan et al. demonstrated the significance of HU density within psoas muscle in predicting mortality rates in patients undergoing transcatheter aortic valve replacement. Psoas density < 25 HU remained significantly associated with mortality at 90 days, one year, and three years, even after controlling for cross-sectional area [14].

Regardless of its precise definition, sarcopenia holds promise as a superior perioperative risk predictor. Its value lies in its independence from the specific surgical procedure and its accessibility from routine preoperative imaging. Its association of LOS with TSMV was not explained by any reduction in postoperative complications. This may mean that it may have some intrinsic value as a predictor of how well a patient will do. It is not readily evident that the thoracic sarcopenia could be a preoperative modifiable factor or is just an indicator of some secondary metric like heart failure, nutritional status, or overall frailty. Prehabilitation is a concept mainly within adult surgery to target intervenable behavioral or lifestyle risk factors in an effort to make a patient more resolute during the perioperative period [15]. For instance, Laohachai et al. reported improved outcomes in adolescent participants with Fontan circulation following 6 weeks of inspiratory muscle training. The training was associated with improved inspiratory muscle strength, resting cardiac output, and ventilatory efficiency of exercise [16]. In a systematic review of studies looking at physical exercise training programs in children and young adults with congenital heart disease out of the immediate perioperative setting, a strong majority of 23 studies (72%) found a significant positive change in activity levels and muscle strength [17]. While exercise training programs have shown potential in enhancing outcomes for congenital cardiac disease patients outside the perioperative setting, a more comprehensive exploration is needed to correlate imaging assessments, muscle training regimens, and clinical outcomes.

## Limitations

Firstly, the retrospective design of our study inherently carries potential biases, most notably the selection bias stemming from the decision-making process behind which patients underwent preoperative imaging. It’s noteworthy that a significant 88.7% of the total index case volume was excluded, which may impact the generalizability of our results. Secondly, the decision to include CT and MRI conducted up to 6 months prior to the index operation may be questionable. Given the relative brevity of pediatric life spans compared to adults, this duration might represent a more substantial and potentially variable period in terms of physiological and anatomical changes. Lastly, our categorization into low and high TSMV groups was based on data from our center-specific patient population. This approach, while pragmatic, may limit the external validity of our findings.

## Conclusion

Our findings indicate that the thoracic musculature holds an association with LOS and mortality in patients undergoing surgery for congenital cardiac anomalies. However, it does not exhibit a discernible correlation with other postoperative morbidities. As such, thoracic musculature merits consideration as a potential risk factor in the preoperative assessment of patients presenting for congenital cardiac surgical interventions.

## Data Availability

The data is not available to keep the privacy of subjects protected.
